# Poplar Propolis Improves Insulin Homeostasis in Non-Diabetic Insulin-Resistant Volunteers with Obesity: A Crossover Randomized Controlled Trial

**DOI:** 10.3390/antiox12081481

**Published:** 2023-07-25

**Authors:** Lea Sani, Nicolas Cardinault, Julien Astier, Patrice Darmon, Jean François Landrier

**Affiliations:** 1 Centre for Nutrition and Cardiovascular Disease (C2VN), INSERM, INRAE, AIX Marseille University, 13000 Marseille, France; lea.sani@univ-amu.fr (L.S.); julien.astier@univ-amu.fr (J.A.); patrice.darmon@ap-hm.fr (P.D.); 2 Association Francophone d’Apithérapie, 58300 Decize, France; nicolas.cardinault@gmail.com

**Keywords:** propolis, insulin resistance, type 2 diabetes mellitus, glucose homeostasis, polyphenols, insulin secretion, oral glucose tolerance test, preventive nutrition, insulin sensitivity Matsuda index

## Abstract

Propolis, a natural resinous mixture rich in polyphenols, produced by bees from a variety of plant sources, has shown significant therapeutic effects and may prevent the development of certain chronic diseases like type 2 diabetes mellitus (T2DM). The objective of this study was to evaluate the effect of supplementation with standardized poplar propolis extract powder (PPEP) on insulin homeostasis in non-diabetic insulin-resistant volunteers with obesity. In this randomized, controlled, crossover trial, nine non-diabetic insulin-resistant volunteers with obesity, aged 49 ± 7 years, were subjected to two periods of supplementation (placebo and PPEP) for 3 months. Blood samples and anthropomorphic data were collected at baseline and at the end of each phase of the intervention. PPEP supplementation improved insulin sensitivity by significantly decreasing the percentage of insulin-resistant subjects and the insulin sensitivity Matsuda index (ISI-M). According to this study, supplementation with standardized PPEP for 3 months in non-diabetic insulin-resistant volunteers with obesity led to an improvement in insulin homeostasis by its effect on insulin resistance and secretion. This study suggests that poplar propolis has a preventive effect on the physiopathological mechanisms of T2DM and, therefore, that it can help to prevent the development of the disease.

## 1. Introduction

Diabetes mellitus, defined as a chronic elevation in the concentration of glucose in the blood, represents a major public health problem, which constitutes a real global pandemic, with an increasing prevalence, affecting more than 537 million adults in 2021 [1]. Type 2 diabetes mellitus (T2DM) is the most common form, reaching 80 to 90% of diabetes cases and particularly affecting the adult population. This disease results in impairments in insulin secretion and insulin action, or both. Its clinical form demonstrates its multifactorial origin: it develops under the influence of genetic and environmental factors, and overweight or obesity plays a major role in its occurrence [2]. During the natural course of T2DM, the expansion of adipose tissue results in a defect in insulin sensitivity (i.e., insulin resistance), with decreased glucose uptake and increased hepatic glucose production, leading to fasting hyperglycemia. In response, there is pancreatic insulin hypersecretion, which defines the asymptomatic phase of prediabetes, resulting in early metabolic abnormalities [3]. This asymptomatic phase is crucial because if prediabetes is left untreated, the risk of developing T2DM is 37% over the next few years, whereas lifestyle intervention could reduce this risk to 20% [4].

Insulin resistance develops in skeletal muscle, liver, adipose tissue, and heart and therefore represents a major cardiovascular risk factor. It is the most powerful predictor of the future development of T2DM and thus represents a therapeutic target in the management of diabetes. Insulin resistance can be easily evaluated by the homeostasis model assessment of insulin resistance (HOMA-IR) index, which includes fasting insulin and glucose values, but a more accurate assessment can be obtained after an oral glucose tolerance test (OGTT) with the Matsuda index, which takes kinetic values into consideration [5]. If the establishment of insulinopenia requires insulin therapy, management of T2DM is primarily based on a healthy, balanced diet and regular physical activity.

Over the past two decades, evidence has been converging in favor of the potential role of specific nutrients in the prevention and management of T2DM or other diseases [6,7,8,9,10]. In particular, micronutrient approaches have been shown to improve insulin sensitivity. Many foods rich in polyphenols have been studied for their preventive effect on the development of T2DM, such as propolis. Propolis is a resinous substance produced by bees from a mixture of harvested resins of various buds from different plant species, beeswax, and their secretions. Propolis is usually used in medicine for its antimicrobial, anti-inflammatory, antitumor, and antioxidant properties, and for its immunomodulatory activity. Overall, propolis is composed of 50% resin and plant balsam, 30% beeswax, 10% essential and aromatic oils, 5% bee pollen, and 5% other mineral and organic materials (sugars, vitamins, minerals, and enzymes) [11]. The chemical composition of propolis depends on its plant source. Poplar propolis is the most studied substance, due to its high polyphenol composition. It is found in temperate zones (Europe, North America, and the non-tropical regions of Asia) and has been characterized by phenolic taxonomic markers (pinocembrin, chrysin, and galangin) and substituted cinnamic acid esters, such as caffeic acid phenylethyl ester (CAPE) [12,13]. The content of biologically active substances is estimated at 70% of the total mass of propolis, including 58% phenolic compounds [14]. Polyphenols are reputed to have a preventive effect on chronic diseases (cancer, cardiovascular diseases, and T2DM) owing to their antioxidant properties [15]. Several preclinical animal studies have proved the efficacy of propolis on glucose, lipid metabolism, insulin, and antioxidant activity [13,16,17,18]. Randomized controlled trials suggest that propolis supplementation in diabetic patients decreases fasting blood glucose and glycated hemoglobin (HbA1c) and has a beneficial effect on the long-term management of T2DM, by reducing oxidative stress and inflammation [19]. However, there are few clinical studies investigating the effect of standardized poplar propolis extract powder (PPEP) on glucose homeostasis in an insulin-resistant population. Accordingly, the present study aimed to evaluate the effect of a specific dose of total polyphenols from poplar propolis on glucose homeostasis and indicators of insulin resistance in non-diabetic insulin-resistant volunteers with obesity.

## 2. Materials and Methods

### 2.1. Ethical Statement

The study was conducted according to the guidelines laid down in the Declaration of Helsinki of 1975 as revised in 2013, and the guidelines for Good Clinical Practice of the ICH. Ethical approval for the involvement of human subjects in this study was granted by the CCP Ouest III Ethics Committee (France), reference number 9.03.20/SI CNRIPH 19.02.11.73507, on 19 April 2019, and registered in ClinicalTrials (NCT05717881).

### 2.2. Study Design

The present trial was a randomized, double-blind, controlled, crossover, dietary intervention study, conducted at the clinal investigation center, Hôpital de la Conception, Marseille, and performed between May 2019 and July 2020. During this trial, two types of supplementations were randomly administered during two treatment periods (placebo and PPEP), using a random number table, in a double-blind manner. The placebo served as the reference group for comparison (control group). Participants and caregivers were blinded to the type of treatment consumed. Each supplementation period lasted 3 months, with a 2-week washout period, to allow the total excretion of polyphenols by the body and not interfere with the new supplementation phase. The subjects in this study were submitted to five visits, allowing the tracking of biological parameters (clinical examination, fasting blood samples, and OGTT) during the study. During the supplementation phases, follow-up telephone calls were carried out in the middle of each supplementation period. Compliance was assessed on a declarative basis of the actual number of pills taken compared to the recommended number of pills calculated on an individual weight basis. PPEP was provided by the Pollenergie company. The polyphenol profile was determined by high-performance liquid chromatography (HPLC) as previously reported by Gardana et al. [20] for propolis powder. The total polyphenol content and the detailed composition of the polyphenols present in poplar propolis powder are reported in Table 1. 

The powder used in this study fully complies with European regulations on food supplements and the more specific one on propolis. Supplements from both groups containing 250 mg of the product were packaged in marine capsules (organic fish gelatin with traces of soy lecithin) and were presented in the same packaging to give them an identical appearance and taste. Supplements in the PPEP group were composed of poplar propolis powder (70% propolis concentrate, 15% magnesium stearate, 10% silicium dioxide, and 5% carob powder), concentrated to 30% total polyphenols. Capsules in the control group contained a placebo powder (92% maltodextrin, 6% magnesium stearate, and 2% silicium dioxide). Subjects were directed not to change their dietary habits, lifestyle, and level of physical activity for the period of the experiment. Subjects in the PPEP group received a dose of propolis adjusted to attain 6 mg total polyphenols/kg body weight, based on the results of a previous preclinical study in mice [16,21]. According to patient weight, six to nine capsules of each supplement were prescribed for oral administration per day.

### 2.3. Participants

**Figure 1:**
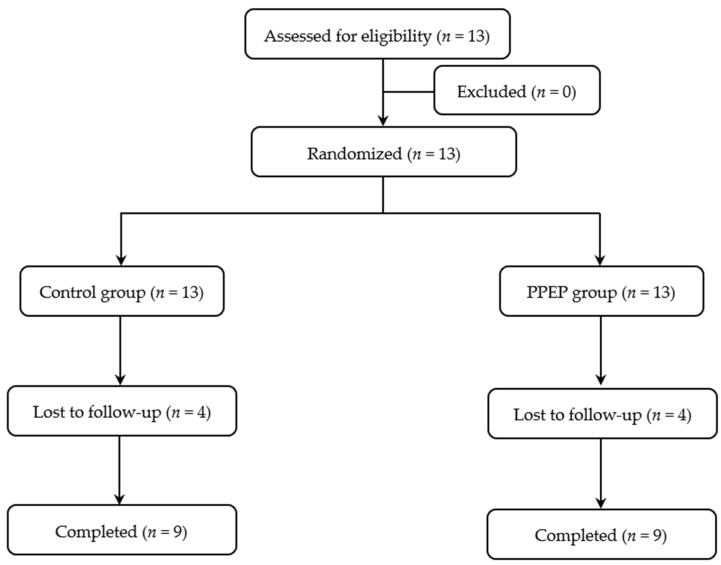
Study participant flow diagram.

The volunteers included were adults under 60 years old with obesity, defined as a body mass index (BMI) ≥ 30 kg/m^2^ [22], as well as insulin-resistant, defined as a HOMA-IR index > 1.85 for men and >2.07 for women [23]. All subjects with any of the following criteria were excluded from the study: the presence of diagnosed diabetes, recent weight change (≥5% in the last 3 months), documented allergy to bee products and/or fish products, positive serology for human immunodeficiency virus or hepatitis, high blood pressure, elevated transaminases (AST > 40 IU/L; ALT > 45 IU/L), low creatinine clearance (estimated glomerular filtration rate < 90 mL/min), interfering treatment (cholesterol-lowering treatment, intestinal absorption modulating treatment, absorption modulating treatment, and/or insulin sensitivity), gastrointestinal tract surgery, pregnancy, and lactation. The subject recruitment flow diagram for this study is presented in Figure 1.

### 2.4. Dietary Survey 

In this study, a dietary assessment based on a 3-day dietary record (portions of food consumed in grams) was performed at the time of subjects’ inclusion to study food habits and to compare dietary energy and nutrient intakes between volunteers. The amount of ingested foods was estimated with the aid of a dedicated picture book [24] and was registered on Nutrilog (nutrilog.com), the reference software for healthcare professionals, which provides a detailed report of nutritional intakes of energy, macro- and micro-nutrients, and alcohol.

### 2.5. Outcomes Measurements

The primary outcome was the difference between pre- and post-supplementation values (Δ) of the insulin sensitivity Matsuda index (ISI-M) between the PPEP and control groups. Secondary endpoints were the difference between the PPEP and control groups (inter-group analysis) and the difference between the baseline and after 3 months of supplementation for each group (intra-group analysis) of other health parameters; glycemia and insulinemia in the fasting state and during OGTT, HbA1c, diabetes indices of insulin sensitivity (HOMA-IR and Si[SI]HGPO), insulin secretion (IGI and AUC_Ins_/AUC_Glu_) and pancreatic beta cell function (HOMA-β and ISSI-2), renal function (creatinine and eGFR), liver function (AST, ALT, AST/ALT ratio, and GTT) lipid profile (total cholesterol, LDL-c, HDL-c, and triglycerides), adipose function (leptin and adiponectin), oxidative stress (8-iso-PGF 2α), and anthropometric data (BMI, waist circumference, body fat mass, and body lean mass).

### 2.6. Oral Glucose Tolerance Test (OGTT)

After each 3-month supplementation period, an OGTT was performed after a 12-h fasting period. Glucose overload with a concentrated glucose solution (75 g of glucose) was performed, and blood samples were collected at fasting and at 30, 60, 90, and 120 min post-overload using a forearm catheter to measure plasma glucose and insulin concentrations.

### 2.7. Measure of Insulin Sensitivity, Insulin Secretion, and Beta Cell Function 

Insulin sensitivity and insulin secretion were assessed using OGTT measurements and through diabetological index calculations, after each supplementation period. Insulin sensitivity was estimated by the ISI-M index, as proposed by Matsuda and DeFronzo (1) [5,25].

(1) $$ISI\sim M=\frac{10,000}{\sqrt{\left[({Glu}_{T0}\times {Ins}_{T0})\times ({Glu}_{mean}\times {Ins}_{mean})\right]}}$$

Insulin sensitivity indices were also calculated by HOMA-IR (2) [26] and simple index assessing insulin sensitivity (SI(is)-OGTT) (3) [27].

(2) $$HOMA\sim IR=\frac{\left({Glu}_{T0}\times {Ins}_{T0}\right)}{22.5}$$

(3) $$SI\left(is\right)\sim OGTT=\frac{1}{\left({\log Glu}_{T0+30+60+90+120}+{\log Ins}_{T0+30+60+90+120}\right)}$$

Insulin secretion indices were calculated by the insulinogenic index (*IGI*) (4) [28] and AUC_Ins_/AUC_Glu_ ratio [29].

(4) $$IGI=\frac{\left({Ins}_{T120}-{Ins}_{T0}\right)}{\left({Glu}_{T120}-{Glu}_{T0}\right)}$$

Beta cell function was estimated by the insulin secretion-sensitivity index (ISSI-2) (5) [5] and homeostasis model assessment of beta cell function (HOMA-β) (6) [26].

(5) $$ISSI\sim 2=\frac{{AUC}_{Ins}}{{AUC}_{Glu}}xISI\sim M$$

(6) $$HOMA\sim \beta =\frac{{(Ins\;}_{T0}\times 20)}{{(Glu\;}_{T0}-3.5)}$$

Volunteers were classified as insulin resistant if the HOMA-IR index was higher than 1.85 for men and 2.07 for women [23].

### 2.8. Measure of Biochemical Parameters 

Biochemical analyses were carried out on blood samples after each supplementation period. After blood sampling and OGTT, samples were collected in heparinized tubes, then centrifuged to obtain plasma. The plasma was then divided into 1.2 mL aliquots and stored at −80 °C. Measurements of creatinine, lipid profile, adipokines, transaminases, and C-reactive protein were performed in the biochemistry department of the Hôpital de la Conception. Serum concentrations of glucose, insulin, and 8-iso-prostaglandin F2α (8-iso-PGF 2α) were determined using commercial colorimetric or enzyme-linked immunosorbent assay (ELISA) kits (Glucose GOD-PAP^®^ Biolabo, Maizy, France; Insulin ELISA, ALPCO Diagnostics^®^, Salem, US, and 8-iso-PGF 2α ELISA, ENZO Life Sciences^®^, Villeurbanne, France, respectively). The kits were used in accordance with the manufacturer’s instructions.

### 2.9. Measure of Anthropometric Parameters 

After each 3-month supplement, anthropometric parameters such as weight, height, waist circumference, and body composition were measured. Weight was measured with light clothing and without shoes, and height without shoes. From these measurements, BMI was calculated by dividing weight (kg) by the square of height (m). Waist circumference (cm) was measured using a tape measure, at the midpoint between the costal margin and the iliac crest, with the subject standing at the end of an exhalation. Body composition was measured using an impedance method (Bodystat 1500, Douglas, British Isles) device, to determine fat mass composition and lean mass composition (%).

### 2.10. Statistical Analysis 

Quantitative and qualitative variables were represented as mean ± SD and percentage prevalence, respectively. The total area under the curve (AUC) of insulin and glucose concentrations during OGTT were calculated for comparison. The normality of data for each group was verified graphically and by the Shapiro–Wilk test. 

The inter-group analysis compared the variations (Δ) between pre- and post-supplementation periods between the control and PPEP groups, according to the following formula: [post-supplementation value − pre-supplementation value], between the control and PPEP groups. Intra-group analysis compared the periods before (baseline) and after (3 months) supplementation for each group (PPEP and control). All comparisons were performed on matched data. Quantitative variables were compared by a Wilcoxon matched-pairs signed rank test or by a paired *t*-test according to data normality. For dichotomous categorical variables, the McNemar test was employed to compare two matched proportions. 

The influence of age was tested before and after each supplementation period using an ANOVA. The regression coefficient (β), the coefficient of determination (R^2^), and the *p*-value (*p*) were calculated for each analysis.

The sample size (*n* = 9 per group for a total of 18 volunteers) was determined, with the variation between baseline and 3 months post-supplementation in the ISI-M as the primary outcome of the study. The calculation assumes a test power (β) of 90% and a significance level (α) of 0.05 to detect a difference of approximately 0.5 unit in the ISI-M (based on the mean and SD of 5.2 ± 0.35) [30], using the following formula (7) [31]:

(7) n=T2n−2−11−α2+T2n−2−11−β2σ^m22εR2

Because the expected dropout rate was 10%, nine participants per supplementation were recruited, i.e., nine non-diabetic insulin-resistant volunteers with obesity who were allocated to the control and PPEP group, leading to a total of 18 subjects. Inter-group and intra-group analyses were performed using Prism (version 9.4.0). The one-way ANOVA was performed using R Studio (version 2022.02.0). A statistically significant value of *p* < 0.05 was used.

## 3. Results

### 3.1. Population Characteristics 

In this study, nine non-diabetic insulin-resistant volunteers with obesity, including eight women and one man (ratio of 1:8), were enrolled in the population, with a mean age of 49 ± 7 years (distribution range of 34 to 58 years). The compliance means were 84.0 ± 21.0% and 95.3 ± 4.6%, respectively, for the PPEP and control groups.

### 3.2. Effect of Poplar Propolis on Insulin Resistance (ISI-M), Insulin Secretion, and Pancreatic Beta Cell Function

**Figure 2:**
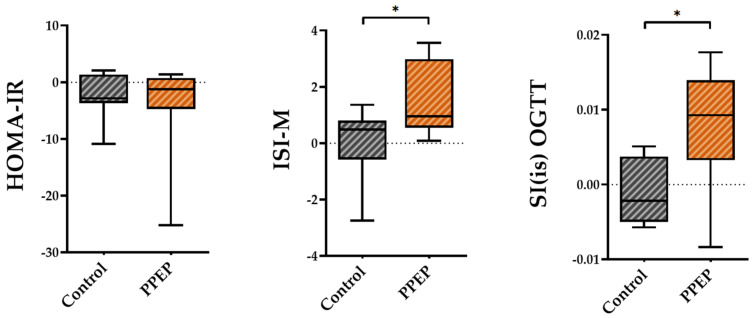
Intergroup comparison of variations in insulin sensitivity indexes between the control and PPEP groups. The variations were calculated using the following formula: (3 months post-supplementation − baseline). Comparison between groups was performed using a paired *t*-test for normally distributed data and a Wilcoxon test for non-normally distributed data; *p* < 0.05 indicates a significant difference. The difference between groups is indicated by *: *p* < 0.05. HOMA-IR, homeostasis model assessment of insulin resistance; ISI-M, insulin sensitivity Matsuda index; SI(is)-OGTT, simple index assessing insulin sensitivity.

The variations between baseline and 3 months post-supplementation in the ISI-M and SI(is)-OGTT insulin sensitivity indexes were significantly higher in the PPEP group than in the control group (1.48 ± 1.3 vs. −0.03 ± 1.2, *p* = 0.02 and 0.008 ± 0.008 vs. −0.001 ± 0.004, *p* = 0.03; Figure 2). By contrast, the variations in the insulin sensitivity index HOMA-IR showed no significant difference between the PPEP and control groups (−4.1 ± 8.3 vs. −2.2 ± 4.0, *p* = 0.5; Figure 2).

**Figure 3:**
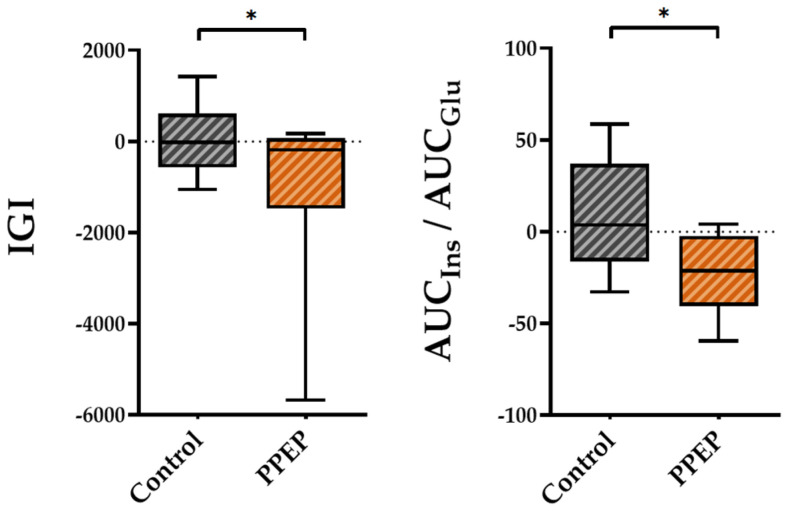
Intergroup comparison of variations in insulin secretion indexes between the control and PPEP groups. The variations were calculated using the following formula: (3 months post-supplementation − baseline). Comparison between groups was performed using a paired *t*-test for normally distributed data and a Wilcoxon test for non-normally distributed data; *p* < 0.05 indicates a significant difference. The difference between groups is indicated by *: *p* < 0.05. AUC, area under the curve; Glu, glucose; IGI, insulinogenic index; Ins, insulin.

Regarding insulin secretion, the variations between baseline and 3 months post-supplementation in the IGI and AUC_Ins_/AUC_Glu_ ratio were lower in the PPEP group than in the control group (−1007 ± 1875 vs. 54.15 ± 778.3, *p* = 0.04 and −22.4 ± 21.4 vs. 9.3 ± 30.8, *p* = 0.03, respectively; Figure 3).

**Figure 4:**
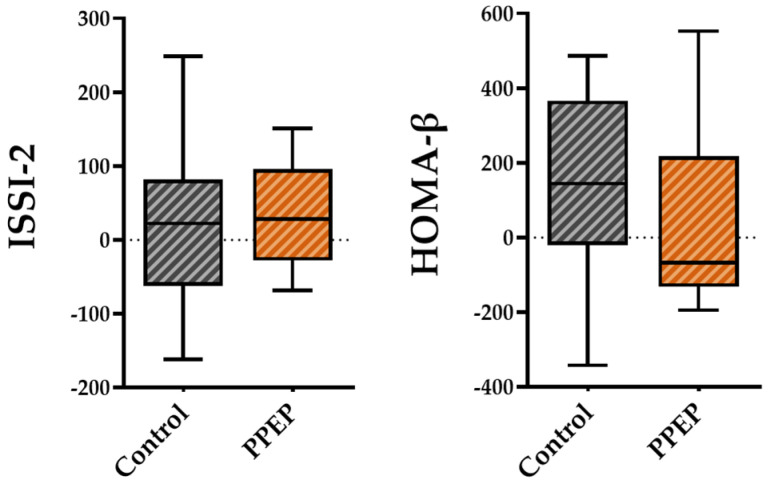
Intergroup comparison of variations in pancreatic beta cell function indexes between the control and PPEP groups. The variations were calculated using the following formula: (3 months post-supplementation − baseline). Comparison between groups was performed using a paired *t*-test for normally distributed data and a Wilcoxon test for non-normally distributed data. ISSI-2, insulin secretion-sensitivity index-2; HOMA-β, homeostasis model assessment of beta cell function.

For pancreatic beta cell function, the variations between baseline and 3 months post-supplementation in the ISSI-2 and HOMA-β indices were not significantly different between the PPEP and control groups (31.6 ± 72.9 vs. 20.1 ± 117.8, *p* = 0.42 and 38.6 ± 253.1 vs. 130.3 ± 261, respectively; Figure 4).

### 3.3. Effect of Poplar Propolis on Glucose and Insulin Homeostasis during OGTT

**Figure 5:**
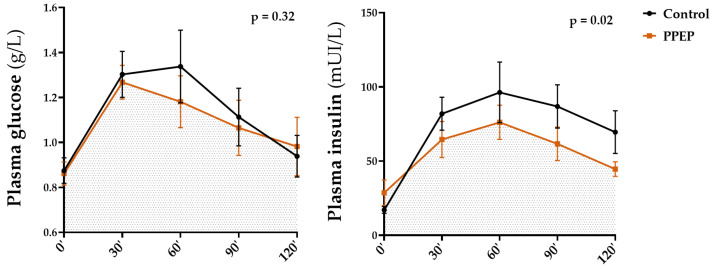
Inter-group comparison of glycemic and insulin responses to OGTT at 3 months post-supplementation between the control and PPEP groups. Data are mean ± standard error of the mean (SEM). Comparison of the AUC of the curves of both groups was performed on paired data by a Wilcoxon matched-pairs signed rank test or by a paired *t*-test depending on the normality of the data; *p* < 0.05 indicates a significant difference.

The values of insulin and glucose plasma concentrations during OGTT in each study group are summarized in Table 2 and Table 3, respectively. Figure 5 shows the comparison of AUCs for glycemia and insulinemia during OGTT, 3 months after each supplementation. The values of plasma concentrations in the fasting state, during OGTT, as well as minimum and maximum values of insulin and glucose were not significantly different between the baseline and after 3 months of supplementation periods (intra-group analysis) and between the PPEP and control groups (inter-group analysis) (Table 2 and Table 3).

Intra-group analysis showed that the mean of insulinemia during OGTT was significantly lower 3 months after PPEP supplementation, in contrast to the control group (79.9 ± 35.0 mUI/L vs. 55.1 ± 17.4 mUI/L, *p* = 0.02 and 68.5 ± 30.5 mUI/L vs. 70.3 ± 31.1 mUI/L, *p* = 0.82; Table 2). Inter-group analysis showed no significant differences between the variations between baseline and 3 months post-supplementation of the PPEP and control groups (−24.7 ± 32.4 mUI/L vs. 1.8 ± 22.6 mUI/L, *p* = 0.08; Table 2). Intra- and inter-group comparison in the AUCs of glycemia during OGTT showed no significant differences between baseline and 3 months after supplementation for the PPEP and control groups (142.9 ± 36.5 vs. 133.1 ± 32.8, *p* = 0.09 and 142.3 ± 43.7 vs. 139.8 ± 38.5, *p* = 0.61; Table 3), between the variations between baseline and 3 months post-supplementation of the PPEP and control groups (−9.8 ± 17.4 vs. −2.5 ± 14.0, *p* = 0.24; Table 3), and between the values at 3 months post-supplementation for each group (*p* = 0.32; Figure 5).

Intra-group analysis of the PPEP group showed a significant 31.0% decrease in the AUCs of insulinemia during the OGTT after 3 months of supplementation (10,704 ± 4718 vs. 6377 ± 1893, *p* = 0.01; Table 2), in contrast to the control group (8933 ± 4109 vs. 10,270 ± 4772, *p* = 0.32; Table 2). The inter-group analysis also showed a significant difference between the variations between baseline and 3 months post-supplementation of the AUCs during OGTT between the PPEP and control groups, with a larger, negative variation for the PPEP group (−4327 ± 4424 vs. 1336 ± 3783, *p* = 0.04; Table 2).

Values at 3 months post-supplementation showed a significantly lower AUC of insulinemia during OGTT in the PPEP group compared with the control group (*p* = 0.02; Figure 4). Moreover, the proportion of insulin-resistant volunteers, based on the HOMA-IR index, was significantly reduced in the PPEP group (99.9% vs. 66.6%, *p* = 0.04; Table 2).

Inter-group analysis showed that the HbA1c variations between baseline and 3 months post-supplementation were significantly different between the PPEP and control groups (−0.02 ± 0.1 vs. 0.22 ± 0.4, *p* = 0.04; Table 3). Furthermore, HbA1c values after 3 months of supplementation in the PPEP and control groups were not significantly different from baseline in the intra-group analysis (5.54 ± 0.5 vs. 5.52 ± 0.5%, *p* = 0.75 and 5.47 ± 0.5 vs. 5.69 ± 0.6%, *p* = 0.40; Table 3).

### 3.4. Effect of Poplar Propolis on Anthropometric and Biological Parameters

As shown in Table 4 and Table 5, which compare both groups, no significant differences regarding anthropometric data (Table 4) or biological parameters (Table 5) were observed between the PPEP and control groups, between baseline and 3 months post-supplementation (intra-group analysis), or between baseline and 3 months post-supplementation variations between the two groups (inter-group analysis). The ratio of energy expended/intake (E/I ratio) was not significantly different between the two groups during the two supplementations (1.52 ± 0.4 vs. 1.60 ± 0.5 kcal/J, *p* = 0.64; Table 4).

## 4. Discussion

The present study examined the efficacy of standardized PPEP supplementation equivalent to 6 mg total polyphenols/kg, for three months, in an insulin-resistant population. To our knowledge, this is the first study to investigate the effect of propolis in non-diabetic insulin-resistant volunteers with obesity. This criterion of judgment on insulin resistance allows us to evaluate the preventive effect of propolis on the development of T2DM. Moreover, in contrast to the usual clinical trials, the methodology of this study adapted the propolis supplementation dosage individually. Although imperfect compliance with daily capsule intake was observed in both groups, this adapted dosage criterion confers reproducibility to this study, enabling the doses tested to be used clinically.

Several factors influence the metabolism of polyphenols, and consequently their bioavailability and excretion time within the body. These factors include the nature of the polyphenols, the quantity consumed, the matrix of the form consumed, the composition of the intestinal microbiota, enzymatic activity, and the composition of the meal ingested. The half-life of polyphenols, i.e., the time required to reduce their concentration in the body by 50%, varies from a few hours to several days [32]. Therefore, a washout period of two months is appropriate to allow the total excretion of the bioactive compounds of propolis, and thereby eliminate the residual effects of supplementation.

The composition of the supplement capsules in the two groups differed for the reason of formulation. Maltodextrin is a poorly digestible carbohydrate manufactured using hydrolysis, purification, and spray-drying methods applied to a variety of starches, and is classically used as a clinical trial placebo. However, it is possible that this supplementation had an effect on the parameters measured in this study. Despite these limitations, the doses of maltodextrin consumed were very low (maximum 2 g per day) and would not be sufficient to impact glucose and insulin homeostasis [33]. Carob powder is derived from the fruit of the Mediterranean carob tree and represents an interesting source of fiber, vitamins, and polyphenols. It has been associated with positive effects on metabolic health, and on glucose homeostasis in particular [34]. Nevertheless, the proportion of carob powder in PPEP capsules was 5%, equivalent to 112.5 mg per day, and no effects associated with this very low dose have been reported [34]. Consequently, it can be assumed that the benefits of PPEP supplementation observed in this study are mainly, if not exclusively, due to propolis.

From a statistical point of view, two statistical analyses were carried out, examining the effect of propolis supplementation alone (intra-group analysis) and the effect of propolis supplementation in comparison with a control group, using the variations between baseline and three months post-supplementation (inter-group analysis). The variation calculation allows us to compare the two groups without taking into consideration the differences in baseline values between the two groups. This double analysis is relevant because it highlights the effect of supplementation itself and in comparison with a control group, in order to exclude any potential confusion bias.

In the present study, we reported a beneficial effect of poplar propolis on insulin sensitivity (ISI-M and SI[is]OGTT) and insulin secretion (IGI and AUC_Ins_/AUC_Glu_). We also demonstrated that standardized PPEP supplementation significantly reduced the proportion of non-diabetic insulin-resistant subjects. This suggests that poplar propolis may improve insulin secretion and insulin sensitivity in insulin-resistant people. Interestingly, these differences are not attributed to lower BMI, body fat, or waist circumference. A meta-analysis conducted on 14 clinical trials revealed that propolis administration led to a significant decrease in insulin resistance (weighted mean difference (WMD): −0.60; 95% confidence interval (CI): −1.20, 0.00) [35]. However, the effect of propolis on insulin resistance, insulin sensitivity, and pancreatic beta cell function has been understudied in clinical trials [36,37,38,39,40]. Furthermore, in these studies, insulin resistance was investigated by HOMA-IR, in contrast to our study which included ISI-M as the primary outcome, which has the advantage of considering kinetic glucose values and has a better correlation with the insulin sensitivity value of peripheral tissue [41]. Only one clinical study studied the effect of propolis on the Matsuda index and showed that a supplementation of 300 mg of propolis with an unknown concentration of polyphenols, in 36 diabetic patients, made it possible to decrease the levels of glycemia in fasting, two hours after OGTT, the AUC of insulin as well as the ISI-M index [42]. Our study also has the advantage of studying multiple diabetic indexes with complementary advantages, which bring convergent information. Additionally, the inter-group analysis also revealed a reduction in total insulinemia AUC during OGTT (*p* = 0.04), also observed in the intra-group analysis for the PPEP group, with an almost 50% reduction between the baseline and three months post-supplementation periods (*p* = 0.01). Mean insulin levels during OGTT were significantly reduced by 31% after supplementation in the PPEP group (*p* = 0.02) (intra-group analysis), although inter-group analysis only showed this tendency in the comparison between the PPEP and control groups (*p* = 0.08). In agreement with our findings, several clinical studies investigating propolis supplementation in a T2DM population, including one using poplar propolis [36], reported that supplementation ranging from 300 to 1500 mg of propolis per day for 8 to 12 weeks, but with an unknown polyphenol concentration, reduced fasting or 2 h postprandial insulin levels, or the AUC of insulin [37,42].

Regarding glucose homeostasis, we observed a difference in the timing of peak blood glucose levels during OGTT between the PPEP and control groups. Whereas the PPEP group reached maximum blood glucose at time T30, the control group peaked at time T60. Moreover, there was also a decreasing trend in glucose levels at OGTT time T60 for the PPEP group compared with the control group (*p* = 0.06). It has recently been shown that glucose levels at 1 h post-OGTT represent a more relevant predictive tool compared to glucose levels at 2 h post-OGTT or total OGTT AUC, for identifying adults at risk of developing T2DM [43,44]. There was a strong tendency for fasting blood glucose levels to decrease between baseline and after three months of PPEP supplementation in the intra-group analysis (*p* = 0.05), but this remained non-significant and was not significantly different from the variation between baseline and after supplementation of the control group in the inter-group analysis (*p* = 0.82).

Furthermore, although intra-group analysis showed no effect of PPEP supplementation on HbA1c levels, it resulted in a non-significant reduction of 0.4% (*p* = 0.75) versus a non-significant increase of 4.1% in the control group (*p* = 0.40). Considering these variation differences, the inter-group analysis showed a significantly reduced HbA1c variation between baseline and three months post-supplementation in the PPEP group compared with the control group (*p* = 0.04). HbA1c is a product of early glycation induced by hyperglycemia in T2DM and its accumulation in red blood cells reveals the average level of glucose to which the cell has been exposed during its life cycle. HbA1c is proportional to the average blood glucose level over the past three months and is an important marker of the risk of hypoglycemia and microvascular complications [45]. According to the epidemiological United Kingdom Prospective Diabetes Study (UKPDS), a 1% reduction in HbA1c would lead to a 21% reduction in the risk of T2DM complications and a 35% reduction in microvascular complications [46]. It is noteworthy that our results related to blood glucose are in agreement with the literature [36,37,38,42,47]. Furthermore, a meta-analysis of six clinical trials including 373 participants with T2DM reported significant reductions in fasting blood glucose (−13.51 mg/dL; 95% CI [−24.98; −2.04]) and HbA1c (−0.52%; 95% CI [−0.94; −0.10]) after propolis supplementation, suggesting that propolis supplementation may be beneficial in controlling blood glucose levels in patients with T2DM [48]. The results related to HbA1c were also confirmed by El-Sharkawy et al. who reported that a three- or six-month propolis supplementation period reduced HbA1c by 0.82% and 0.96%, respectively, in T2DM patients with chronic periodontitis [49].

From a molecular point of view, it has been postulated that the main mechanism of glycemic and insulinemic regulation of propolis could be due to the antioxidant activity of flavonoids. As a matter of fact, these flavonoids would enable the scavenging of free radicals causing significant cellular damage [37]. The recent study by Cardinault et al. shows that the polyphenols present in propolis promote the transactivation of nuclear factor (erythroid-derived 2)-like 2 (Nrf2), thus leading to the production of antioxidant enzymes, the prevention of oxidative stress, but also improvement in glucose homeostasis [13,16]. Moreover, these doses of poplar propolis ethanolic extract (PPEE) administered to mice have been standardized (4.5 mg of total polyphenols per mouse per day) and are transposable to the doses in our human study [50]. The hypoglycemic effect of propolis could also be mediated by its positive effect on the phosphorylation and number of insulin receptors [39,51], increased glucose uptake and translocation of the insulin-sensitive GLUT 4 receptor [52], and down-regulation of genes involved in gluconeogenesis pathways. These properties would result in enhanced cell sensitivity to insulin and increased insulin production by pancreatic β-cells [53].

The existence of a positive relationship between age and insulin resistance is well established. Indeed, studies have observed an increase in insulin resistance with age, which may contribute to the development of T2DM [54,55]. In this study, a univariate ANOVA was used to investigate the effect of age on the Matsuda index, which reflects insulin resistance, in each group. This analysis confirmed that age had no influence on insulin resistance before and after supplementation in the PPEP (β = 0.014, R^2^ = 0.003, *p* = 0.87 and β = 0.006, R^2^ = 0.001, *p* = 0.95) and control (β = 0.110, R^2^ = 0.155, *p* = 0.30 and β = 0.050, R^2^ = 0.085, *p* = 0.45) groups.

No effect on lipid profile, adipokine levels, transaminases, BMI, and body composition was reported under propolis supplementation in the present study. It is noteworthy that several studies have shown a beneficial effect of propolis on lipid balance by decreasing the level of triglycerides and LDL-cholesterol [38] as well as increasing the level of HDL-cholesterol [36,39]. Further studies have demonstrated that supplementation with 60 mg of propolis significantly decreased leptin levels in obese patients [56]. A study evaluating the effect of supplementation of 1000 mg of Iranian propolis demonstrated that it decreased the levels of AST and ALT transaminases. Our study also showed that PPEP decreased the level of C-reactive protein (CRP) [36]. Other studies have found a positive effect of propolis on other inflammatory markers [57,58,59,60,61]. None of the clinical studies reported an effect of propolis on BMI or body composition, in contrast to the preclinical studies [13,16,62]. The origin of such discrepancies between our data and other reports is presently unknown and would require further studies. Nevertheless, it is widely assumed that the high variability in the results of clinical studies could be related to the botanical origin and posology in the active principles of the propolis used. A standardization of the propolis used is necessary for future clinical studies. Our study has the advantage of investigating the effect of poplar propolis supplementation and particularly of a PPEP dose equivalent to 6 mg of total polyphenols/kg of body weight, reputed for its phenolic signature leading to significant beneficial effects on health in preclinical studies. Our study also has the advantage of lasting three months, which is sufficient to measure changes in biological parameters, including HbA1c. Longer studies will be necessary to confirm the short-term results, as well as test the efficacy and safety of propolis in the context of insulin resistance. Furthermore, the present study included only nine volunteers, on the basis of a power calculation, so it will be mandatory to expand the number of subjects to validate our hypothesis. Nevertheless, it is important to recall that we implemented a double-blind crossover design, where every volunteer is their own control, which increases the power of the study while reducing volunteer recruitment, but also limiting inter-individual variability, resulting in homogeneous sample groups.

## 5. Conclusions

In conclusion, supplementation with standardized PPEP reaching 6 mg of total polyphenols per kg of body weight for three months regulated insulin homeostasis, as reflected by the improvement in several indices. Poplar propolis supplementation does not replace a healthy, balanced diet, but represents a sustainable alternative treatment strategy for chronic diseases such as T2DM. In fact, it could prevent the development of diabetes in the context of insulin resistance. Finally, other clinical studies are necessary to verify the effect of long-term supplementation of PPEP on the prevention and management of T2DM.

## Figures and Tables

**Table 1:** Polyphenol content of poplar propolis powder used in PPEP supplementation.

Polyphenol Content ^1^	Quantity ^2^
Pinocembrin	3718
Chrysin	2580
Galangin	2006
Caffeic acid phenethyl ester (CAPE)	1782
Coumaric acid	800
Caffeic acid	756
Cinnamic acid	611
Kaempferol	526
Quercetin	414
Ferulic acid	263
Apigenin	198

^1^ Considers only the phenolic composition of propolis and not of other capsule components. ^2^ Quantity in mg/100 g of PPEP.

**Table 2:** Comparative table of insulin homeostasis between the control and PPEP groups.

		Control (*n* = 9)	*p*-Value ^1^	PPEP (*n* = 9)	*p*-Value ^1^	*p*-Value ^2^
Insulin Homeostasis (mUI/L)						
Fasting insulinemia	Baseline	25.2 ± 12.4	0.19	29.9 ± 21.7	0.91	
	3 months	17.1 ± 6.8		28.7 ± 25.6		
	Δ	−8.1 ± 16.2		−1.2 ± 30.1		0.56
Time 30 of OGTT	Baseline	79.3 ± 26.2	0.80	83.1 ± 30.6	0.18	
	3 months	81.9 ± 33.3		64.6 ± 36.4		
	Δ	2.6 ± 29.1		−18.6 ± 38.4		0.17
Time 60 of OGTT	Baseline	90.6 ± 62.5	0.74	110.7 ± 54.2	0.06	
	3 months	96.3 ± 61.4		76.1 ± 34.7		
	Δ	5.7 ± 48.6		−34.6 ± 47.9		0.16
Time 90 of OGTT	Baseline	87.7 ± 48.7	0.93	109.7 ± 77.3	0.10	
	3 months	86.9 ± 43.7		61.7 ± 33.7		
	Δ	−0.8 ± 26.3		−48.0 ± 78		0.13
Time 120 of OGTT	Baseline	59.9 ± 37.6	0.36	65.9 ± 49.8	0.16	
	3 months	69.6 ± 43.1		44.6 ± 14.7		
	Δ	9.7 ± 29.9		−21.3 ± 40.7		0.15
Minimum value	Baseline	23.2 ± 11	0.24	23.2 ± 16	0.41	
	3 months	17.1 ± 6.8		17.1 ± 9.4		
	Δ	−6.0 ± 13		−6.1 ± 17.3		0.99
Maximum value	Baseline	113.8 ± 60.5	0.97	132.5 ± 71.1	0.17	
	3 months	114.5 ± 58.8		95.1 ± 35.1		
	Δ	0.7 ± 47.3		−37.4 ± 74.1		0.49
Mean value	Baseline	68.5 ± 30.5	0.82	79.9 ± 35.0	**0.02**	
	3 months	70.3 ± 31.1		55.1 ± 17.4		
	Δ	1.8 ± 22.6		−24.7 ± 32.4		0.08
AUC insulinemia	Baseline	8933 ± 4109	0.32	10,704 ± 4718	**0.01**	
	3 months	10,270 ± 4772		6377 ± 1893		
	Δ	1336 ± 3783		−4327 ± 4424		**0.04**
Insulin resistance ^3^	Yes	9 (99.9%)		6 (66.6%)		
	No	0 (0.0%)		3 (33.3%)		**0.04**

Intra-group comparison (baseline vs. 3 months after supplementation for each group) and inter-group comparison (variation for PPEP vs. variation for control). ^1^ Intra-group comparison between the pre-supplementation (baseline) and post-supplementation (3 months) values for the PPEP and control groups. ^2^ Inter-group comparison of variations (Δ) between the PPEP and control groups. The variations were calculated using the following formula: (3 months post-supplementation − baseline). ^3^ The threshold of insulin resistance was defined by a HOMA-IR index > 1.85 for men and >2.07 for women. Data are mean ± standard deviation (SD) and quantity (%) for continuous and categorical variables, respectively. Comparison of two groups was performed on matched data by a Wilcoxon matched-pairs signed rank test or by a paired *t*-test according to data normality, *p* < 0.05 indicates a significant difference, highlighted in bold. AUC, area under the curve; OGTT, oral glucose tolerance test; Δ, variation between pre- and post-supplementation values.

**Table 3:** Comparative table of glucose homeostasis between the control and PPEP groups.

		Control (*n* = 9)	*p*-Value ^1^	PPEP (*n* = 9)	*p*-Value ^1^	*p*-Value ^2^
Glucose Homeostasis (g/L)						
HbA1c (%)	Baseline	5.47 ± 0.5	0.40	5.54 ± 0.5	0.75	
	3 months	5.69 ± 0.6		5.52 ± 0.5		
	Δ	0.22 ± 0.4		−0.02 ± 0.1		**0.04**
Fasting glycemia	Baseline	0.97 ± 0.3	0.29	0.97 ± 0.2	0.05	
	3 months	0.87 ± 0.2		0.86 ± 0.2		
	Δ	−0.1 ± 0.2		−0.1 ± 0.2		0.82
Time 30 of OGTT	Baseline	1.4 ± 0.4	0.64	1.3 ± 0.3	0.14	
	3 months	1.3 ± 0.3		1.3 ± 0.2		
	Δ	0.22 ± 0.4		−0.22 ± 0.1		0.91
Time 60 of OGTT	Baseline	1.3 ± 0.5	0.46	1.3 ± 0.4	0.15	
	3 months	1.3 ± 0.5		1.2 ± 0.3		
	Δ	0.05 ± 0.06		−0.11 ± 0.21		0.06
Time 90 of OGTT	Baseline	1.1 ± 0.4	0.99	1.1 ± 0.4	0.09	
	3 months	1.1 ± 0.4		1.1 ± 0.4		
	Δ	0.1 ± 0.2		−0.1 ± 0.2		0.30
Time 120 of OGTT	Baseline	0.90 ± 0.3	0.54	0.97 ± 0.4	0.99	
	3 months	0.94 ± 0.3		0.98 ± 0.4		
	Δ	0.03 ± 0.2		0.01 ± 0.2		0.49
Minimum value	Baseline	0.86 ± 0.3	0.37	0.82 ± 0.2	0.72	
	3 months	0.82 ± 0.2		0.80 ± 0.2		
	Δ	−0.05 ± 0.2		−0.02 ± 0.2		0.69
Maximum value	Baseline	1.5 ± 0.4	0.61	1.4 ± 0.3	0.19	
	3 months	1.4 ± 0.5		1.3 ± 0.3		
	Δ	−0.03 ± 0.2		−0.09 ± 0.2		0.30
Mean value	Baseline	1.14 ± 0.3	0.58	1.15 ± 0.3	0.16	
	3 months	1.11 ± 0.3		1.07 ± 0.3		
	Δ	−0.02 ± 0.1		−0.08 ± 0.1		0.35
AUC glycemia	Baseline	142.3 ± 43.7	0.61	142.9 ± 36.5	0.09	
	3 months	139.8 ± 38.5		133.1 ± 32.8		
	Δ	−2.5 ± 14.0		−9.8 ± 17.4		0.24

Intra-group comparison (baseline vs. 3 months after supplementation for each group) and inter-group comparison (variation for PPEP vs. variation for control). ^1^ Intra-group comparison between pre-supplementation (baseline) and post-supplementation (3 months) values for the PPEP and control groups. ^2^ Inter-group comparison of variations (Δ) between the PPEP and control groups. The variations were calculated using the following formula: (3 months post-supplementation − baseline). Data are mean ± standard deviation (SD) and quantity (%) for continuous and categorical variables, respectively. Comparison of two groups was performed on matched data by a Wilcoxon matched-pairs signed rank test or by a paired *t*-test according to data normality; *p* < 0.05 indicates a significant difference, highlighted in bold. AUC, area under the curve; HbA1c, glycated hemoglobin; OGTT, oral glucose tolerance test; Δ, variation between pre- and post-supplementation values.

**Table 4:** Comparative table of anthropometric parameters between the control and PPEP groups.

		Control (*n* = 9)	*p*-Value ^1^	PPEP (*n* = 9)	*p*-Value ^1^	*p*-Value ^2^
Anthropometric and Nutritional Data						
BMI (kg/m ^2^)	Baseline	31.7 ± 3.2	0.97	31.5 ± 3.2	0.46	
	3 months	31.7 ± 3.1		31.7 ± 3.1		
	Δ	−0.01 ± 0.7		0.21 ± 0.8		0.49
Waist circumference	Baseline	98.7 ± 9.6	0.43	98.1 ± 9.9	0.19	
(cm)	3 months	95.1 ± 16.6		99.3 ± 8.9		
	Δ	−3.6 ± 12.9		1.2 ± 2.5		0.29
Body fat (%)	Baseline	41.1 ± 6.8	0.62	41.2 ± 6.9	0.96	
	3 months	41.4 ± 6.8		41.2 ± 6.6		
	Δ	0.26 ± 1.4		−0.03 ± 2.1		0.99
Lean body mass (%)	Baseline	58.9 ± 6.8	0.62	58.6 ± 6.9	0.85	
	3 months	58.6 ± 6.8		58.8 ± 6.6		
	Δ	−0.27 ± 1.4		0.14 ± 2.3		0.99
E/I ratio ^3^ (kcal/J)	Baseline	1.60 ± 0.5		1.52 ± 0.4		0.64

Intra-group comparison (baseline vs. 3 months after supplementation for each group) and inter-group comparison (variation for PPEP vs. variation for control). ^1^ Intra-group comparison between pre-supplementation (baseline) and post-supplementation (3 months) values for the PPEP and control groups. ^2^ Inter-group comparison of variations (Δ) between the PPEP and control groups. The variations were calculated using the following formula: (3 months post-supplementation − baseline). ^3^ The ratio of energy expended/intake was calculated using the following formula: (total daily energy expenditure_(kcal)_ ÷ calorie intake_(kcal)_) ÷ weight_(kg)_ × 100). Data are mean ± standard deviation (SD). Comparison of two groups was performed on matched data by a Wilcoxon matched-pairs signed rank test or by a paired *t*-test according to data normality; *p* < 0.05 indicates a significant difference. BMI, body mass index; E/I ratio, ratio of energy expended/intake; Δ, variation between pre- and post-supplementation values.

**Table 5:** Comparative table of biological parameters between the control and PPEP groups.

		Control (*n* = 9)	*p*-Value ^1^	PPEP (*n* = 9)	*p*-Value ^1^	*p*-Value ^2^
Biological data						
Renal function data						
Creatinine (µmol/L)	Baseline	66.9 ± 13	0.51	65.2 ± 12.6	0.22	
	3 months	68.1 ± 12.8		63.1 ± 12.9		
	Δ	1.2 ± 5.2		−2.1 ± 4.7		0.57
Creatinine clearance ^3^	Baseline	127.2 ± 43.3	0.41	129.1 ± 43.1	0.16	
	3 months	124.2 ± 41.9		133.3 ± 39.9		
	Δ	−3.1 ± 8.8		4.2 ± 9		0.30
Liver function data						
AST (UI/L)	Baseline	21.4 ± 4.8	0.82	22.8 ± 4.5	0.62	
	3 months	21.2 ± 5		21.9 ± 4.7		
	Δ	−0.2 ± 2.9		−0.9 ± 5.3		0.89
ALT (UI/L)	Baseline	22 ± 12.7	0.99	23.1 ± 12.7	0.55	
	3 months	20.7 ± 8.4		21.8 ± 10.6		
	Δ	−1.3 ± 8.2		−1.3 ± 12.1		0.99
AST/ALT	Baseline	1.2 ± 0.5	0.99	1.3 ± 0.8	0.91	
	3 months	1.2 ± 0.6		1.1 ± 0.4		
	Δ	0.05 ± 0.3		−0.12 ± 0.5		0.91
GGT (UI/L)	Baseline	23.6 ± 12.1	0.83	25. 9 ± 14.9	0.15	
	3 months	21 ± 10.6		24 ± 12.6		
	Δ	−2.6 + 11.9		−1.9 ± 3.5		0.88
Lipid profile data						
Total cholesterol (g/L)	Baseline	2.1 ± 0.4	0.91	2.1 ± 0.3	0.92	
	3 months	2.1 ± 0.4		2.1 ± 0.3		
	Δ	0.03 ± 0.3		0.01 ± 0.3		0.99
Triglycerides (g/L)	Baseline	1.0 ± 0.4	0.76	1.1 ± 0.4	0.75	
	3 months	1.0 ± 0.4		1.1 ± 0.4		
	Δ	0.04 ± 0.4		0.02 ± 0.1		0.73
HDL-cholesterol (g/L)	Baseline	0.6 ± 0.1	0.75	0.6 ± 0.1	0.31	
	3 months	0.6 ± 0.1		0.5 ± 0.1		
	Δ	−0.01 ± 0.08		−0.01 ± 0.04		0.82
LDL-cholesterol (g/L)	Baseline	1.3 ± 0.3	0.77	1.4 ± 0.2	0.75	
	3 months	1.4 ± 0.4		1.4 ± 0.2		
	Δ	0.03 ± 0.2		0.03 ± 0.2		0.91
Adipose tissue function data						
Leptin (pg/mL)	Baseline	42,931 ± 18,137	0.73	37,266 ± 16,592	0.18	
	3 months	44,344 ± 18,395		45,587 ± 24,241		
	Δ	1413 ± 11,710		8321 ± 16,861		0.36
Adiponectin (ng/mL)	Baseline	6882 ± 4462	0.91	7010 ± 4883	0.34	
	3 months	6791 ± 4475		6655 ± 5069		
	Δ	−90.6 ± 1107		−355.4 ± 974		0.20
Oxidative stress data						
8-iso-PGF 2α (pg/mL)	Baseline	1533 ± 2812	0.16	1021 ± 1549	0.34	
	3 months	875 ± 1325		425 ± 393		
	Δ	−659 ± 1512		−595 ± 1626		0.91

Intra-group comparison (baseline vs. 3 months after supplementation for each group) and inter-group comparison (variation for PPEP vs. variation for control). ^1^ Intra-group comparison between pre-supplementation (baseline) and post-supplementation (3 months) values for the PPEP and control groups. ^2^ Inter-group comparison of variations (Δ) between the PPEP and control groups. The variations were calculated using the following formula: (3 months post-supplementation—baseline). ^3^ Creatinine clearance was calculated using the Cockroft and Gault formula. Data are mean ± standard deviation (SD). Comparison of two groups was performed on matched data by a Wilcoxon matched-pairs signed rank test or by a paired *t*-test according to data normality; *p* < 0.05 indicates a significant difference. ALT, alanine aminotransferase; AST, aspartate aminotransferase; GGT, gamma-glutamyl transferase; HDL, high-density lipoprotein; LDL, low-density lipoprotein; 8-iso-PGF 2α, 8-iso-prostaglandin F2α; Δ, variation between pre- and post-supplementation values.

## Data Availability

The datasets analyzed in the current study are not publicly available due to ethical reasons and because our participants only gave their consent for the use of their data by the original team of investigators.
